# Serial Ultrasonographic and Real-Time Elastosonographic Assessment of the Ovine Common Calcaneal Tendon, after an Experimentally Induced Tendinopathy

**DOI:** 10.3390/vetsci8040054

**Published:** 2021-03-25

**Authors:** Daniele Serrani, Antonella Volta, Franco Cingolani, Luca Pennasilico, Caterina Di Bella, Mattia Bonazzi, Alberto Salvaggio, Angela Palumbo Piccionello

**Affiliations:** 1 Southern Counties Veterinary Specialists, Forest Corner Farm, Hangersley, Ringwood, Hampshire BH24 3JW, UK; 2 Dipartimento di Scienze Medico Veterinarie, Università degli studi di Parma, 43100 Parma, Italy; antonella.volta@unipr.it (A.V.); bonazvox@libero.it (M.B.); 3 Scuola di Bioscienze e Medicina Veterinaria, Università degli studi di Camerino, UO di Chirurgia, 62024 Matelica, Italy; franco.cingolani@studenti.unicam.it (F.C.); luca.pennasilico@studenti.unicam.it (L.P.); caterina.dibella@unicam.it (C.D.B.); angela.palumbo@unicam.it (A.P.P.); 4 Clinica Veterinaria San Silvestro, 52043 Castiglion Fiorentino, Italy; alberto.salvaggio@icloud.com

**Keywords:** sheep, sonoelastography, achilles tendon

## Abstract

Real-time elastosonography (RTE) is a recently described, non-invasive, ultrasonographic technique developed to assess tissue elasticity. The main aim of this study was to investigate the ultrasonographic and elastosonographic appearance of the common calcaneal tendon (CCT) in an ovine model, and to monitor the progression of tendon healing after an experimentally-induced tendinopathy. Sound tendons were initially evaluated (T_0_) with a caliper and by a single operator with ultrasound. Ultrasonographic and elastosonographic images were then acquired. Subsequently, ultrasound-guided tendon lesions were induced by injecting 500 IU of Type IA collagenases proximally to the calcaneal tuberosity. Caliper measurement, ultrasonography and elastosonography were then repeated at 15 (T_1_), 30 (T_2_) and 60 (T_3_) days. Clinically measured width of the tendon, ultrasonographic thickness and width and percentage of hard (Elx-t%hrd) and soft (Elx-t%sft) tissue were recorded. Statistical analysis was performed on the data collected; statistical significance was set at *p* < 0.05. Intra-class correlation coefficient (ICC) revealed good (0.68) repeatability of elastosonographic evaluation of the CCT. The tendon width was significantly increased when comparing T_0_ with T_1–2_ and decreased when comparing T_1–2_ with T_3_. Ultrasound-assessed thickness was significantly increased between T_0_–T_1_ and decreased between T_1_-T_2–3_. Elx-t%hrd was significantly decreased at T_1–2–3_ and Elx-t%sft was significantly increased at T_1–2–3_. In conclusion, the ovine CCT is a highly stiff structure that undergoes a severe loss of stiffness during the healing process. Thickness and width of the tendon increased during the first 30 days and then reduced progressively along the subsequent 30 days. Ultrasonographic appearance of the tendon remained severely abnormal and the tendon showed severely reduced elastic proprieties 60 days after lesion induction.

## 1. Introduction

Achilles tendinopathies are one of the most common tendon injuries in human medicine [1] and account for 30–50% of all sports-related injury [2]. Re-rupture of the Achilles tendon following conservative or surgical treatment is a well described complication and is frequently caused by return to sport activity before complete tendon healing [3]. In veterinary medicine, the Achilles tendon, more commonly defined as the common calcaneal tendon (CCT), is composed of the convergence of three separate tendons at the level of the calcaneal tuberosity: The gastrocnemius tendon, the superficial digital flexor tendon (SDFT) and the combined tendons of the biceps femoris, gracilis and semitendinosus [4].

In small animals, although common calcaneal tendon rupture is uncommon, it has frequently been reported [5]. Typically, this condition is degenerative or traumatic in origin. Degenerative disease is more frequently encountered in large-breed dogs, with Labrador Retrievers, Doberman Pinchers and German Shepherds over represented [5,6,7,8]. The gastrocnemius is the most commonly affected component of the CCT [5]. In the horse, common calcaneal tendinopathies have been reported only as a consequence of trauma [9].

The prognosis to return to vigorous athletic activity following CCT rupture in dogs is fair to poor [10]. Ultrasonographic assessment of the CCT healing has been reported in dogs [11]. The hypovascularity of tendons, and its low metabolic rate, results in poor tendon healing capacity and loss of elastic proprieties [1]. Clinical assessment of the elastic proprieties of tendons has become possible in the last decades; however, to the authors knowledge, evaluation of the biomechanical proprieties of the CCT following a tendon injury, have been only reported on a rabbit model [12].

Real-time elastosonography (RTE) is a recently described, non-invasive technique that uses the ultrasound to assess the mechanical proprieties of tissues [13]. RTE allows evaluation of tissue elasticity by applying pressure (stress) on a tissue with the ultrasound probe and comparing the change in shape (strain) with the spatial displacement of it [13,14,15,16]. The strain information is commonly displayed by the elastosonographic software as a color-coded map (elastogram) or as a semi-quantitative strain ratio between two user-defined region of interest (ROI) [17]. In small animals, RTE has been reported to be a repeatable and reproducible technique to assess the patellar ligament in sound dogs [18,19]. In horses, RTE has been reported to be a feasible technique to evaluate sound metacarpal tendons [20] and soft tissue injuries of the distal hindlimb [21]. Moreover, preliminary data suggest that RTE could provide, additional, mechanical information to assess injured superficial digital flexor tendon healing [22].

In the human literature, the use of RTE has been described in the assessment of the musculo-skeletal system [23]. Despite its frequent use, there is still a lack of consensus with regards to the application of this technique when assessing tendon healing. Between the different tendons, RTE has been mostly investigate in the assessment of the Achilles tendon, and is reportedly superior to traditional ultrasound (US) and power doppler (PD) for the evaluation of tendon healing [24]. The sensitivity of RTE to predict signs of histopathologic degeneration of Achilles tendinosis is superior to traditional US [25].

In the last twenty years, considering its low cost, availability and its acceptance by the society as research animal, the ovine specie has become one of the most commonly used large animal model in orthopedic research [26]. The results of these researches have often been the starting point for the diagnosis and treatment of pathologies in humans and many other animal species.

The aim of this study is to evaluate the ultrasonographic and elastosonographic appearance of the common calcaneal tendon in an ovine model and to assess the clinical, ultrasonographic and elastosonographic progression of the tendon healing process after an experimentally-induced tendinopathy.

## 2. Materials and Methods

The data collected for this prospective, longitudinal, experimental study are part of a larger study regarding common calcaneal tendon healing evaluation following stem cells inoculation. Ethical approval was provided by the Italian Ministry of Health (approval n.620/2018-PR).

Twelve clinically healthy female sheep of age between two and five years, and weight between 50 and 60 kg, were prospectively enrolled in this study. Before enrolment, each ovine underwent physical, complete orthopedic and neurologic examinations and hematology and serum biochemical analyses. Inclusion criteria were as follows: Being an American Society of Anaesthesiologist (ASA) Physical Status I, unremarkable orthopedic and neurologic exam, absence of clinicopathological abnormalities, absence of pregnancy (confirmed by ultrasound examination).

Study participants were intramuscularly sedated with midazolam and buprenorphine (0.3 mg/kg and 15 μg/kg, respectively). Participants were positioned in right lateral recumbency with the stifle and tarsus respectively flexed to approximately 135° and 90°, such that the CCT was on a neuter position. The caudal region between stifle and tarsus was shaved. Clinical evaluation of the tendon width was performed with a caliper approximately 6 cm proximally to the CCT insertion. Coupling echographic gel was applied to the skin over the area of interest. Conventional B-mode ultrasonographic examination of the CCT was performed using a My Laboratory Class C ultrasound machine (Esaote; Genova, Italy) equipped with a 3–11 MHz multifrequency linear transducer (LA 533, Esaote Genova, Italy). A standardized scanning protocol with optimized B-mode scanning parameters such as focal zone, depth and frequency was used at each time point. Longitudinal and transverse images were acquired in the middle portion of the tendon, approximately 6 cm proximally to the insertion of the tendon at level of the calcaneal tuberosity.

Tendons that exhibited ultrasonographic evidences of pathology, such as disrupted architecture, internal mineralization or increased cross-sectional diameter, were excluded. Real-time elastosonography was then performed with the same machine equipped with a dedicated software (ElaXto, Esaote, Genoa, Italy) and by a single radiologist. Elastosonographic images were obtained by applying light rhythmic pressure with the probe as previously described by Piccionello PA et al., 2018, and recorded on the ultrasonographic machine. Transducer pressure was applied horizontally, keeping the probe perfectly perpendicular to the CCT to avoid anisotropy, and was adjusted according to the real-time visual marker for compression shown on the screen (a grey spring indicating insufficient or excessive tissue deformation, whereas a green spring indicates appropriate tissue deformation). This marker gives instant feedback regarding the amount of pressure applied and ensure consistency during image acquisition. The same operator evaluated each CCT twice with a pause of 10 min between the examinations. Only the left CCT was examined for this study. The right CCTs were subsequently examined and data collected as part of a different study.

Subsequently, 500 IU of type IA collagenases were injected into the peritendinous space of the CCT under ultrasound guidance, 6 cm proximal to its insertion on the calcaneal tuberosity, using a 22-gauge needle.

The study participants were treated with systemic nonsteroidal anti-inflammatory medications for four days (flunixin meglumine 1 mg/kg IM-Finadyne 5%) and reassessed clinically once daily for 60 days. Clinical, ultrasonographic and elastosonographic tendon assessments were performed at day 15 (T_1_), 30 (T_2_) and 60 (T_3_). Tendon width, clinically assessed with the caliper, was recorded at each time point.

The following ultrasound grading scheme was used to describe the appearance of the CCT [27]:

- Grade 1: Normal tendon with parallel fibers and homogeneous architecture
- Grade 2: Enlarged tendon with bowed margins and homogeneous architecture
- Grade 3: Hypoechoic area with or without tendon enlargement and bowed margins

Tendon thickness and width expressed in mm were recorded on the transverse ultrasound section for each evaluation.

The elastosonographic images were characterized by the superimposition of the elastogram (conventional color translucent map) on the B-mode images with different colors indicating the relative elasticity of the tissues of the ROI examined, compared with the mean elasticity of the entire area. The color map was as follows: Blue (hard tissue), green (intermediate tissue) and red (soft tissue). Only images without artefacts were recorded and stored. A second elastosonographic examination was obtained for assessing repeatability after about 10 min. Following acquisition of all the images, each elastogram was subsequently processed with the aforementioned ElaXto software. For each sample, ROIs were drawn over the entire area in which the tendon lesion was induced, on the elastosonographic images, in order to quantitatively measure the percentage of hard (Elx-t%hrd) and soft (Elx-t%sft) tissue within the tendon.

Statistical analysis was performed on the data collected with the software IBM^®^ SPSS^®^ Amos v.25 (Chicago, IL, USA). Statistical significance was set at *p* < 0.05. The intra-class correlation coefficient (ICC) was calculated for the first and second round of measurements by the same operator (intra-observer) in order to assess repeatability of elastosonographic evaluation of the ovine CCT. An ICC > 0.75 was considered high correlation, an ICC between 0.74 and 0.60 was considered good correlation, an ICC between 0.59 and 0.4 was considered fair correlation, an ICC less than 0.40 was considered poor correlation [28].

The Shapiro–Wilk test was preliminary calculated to evaluate the distribution of the data. Data were not normally distributed. For this reason, Wilcoxon signed-rank test was used to compare clinically assessed tendon width, CCT thickness and width measured with ultrasound, Elx-t%hrd and Elx-t%sft of the tendons in the following groups of data: (1) sound tendons at T_0_ and tendons at T_1_, T_2_ and T_3_, (2) lesioned tendons at T_1_, T_2_ and T_3_ and (3) lesioned tendons at T_2_ and T_3_.

## 3. Results

**Figure 1:**
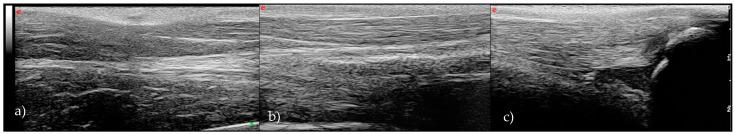
Longitudinal ultrasonographic scan of the normal ovine common calcaneal tendon (CCT): (**a**) myotendinous junction, (**b**) middle part and (**c**) insertion on the calcaneal tuberosity.

Twelve, adult female, clinically healthy sheep, between two and five years of age matched the inclusions criteria. Traditional ultrasonography allowed visualization of the CCT along the entirety of its length in both the longitudinal and transverse planes. There were obvious distinctions between the main components of the tendon; the superficial digital flexor and the gastrocnemius tendon, respectively positioned superficial and deep one to each other. In the longitudinal plane, the CCT was characterized by parallel hyperechoic echotexture (Archambault scheme: Grade 1) (Figure 1).

**Figure 2:**
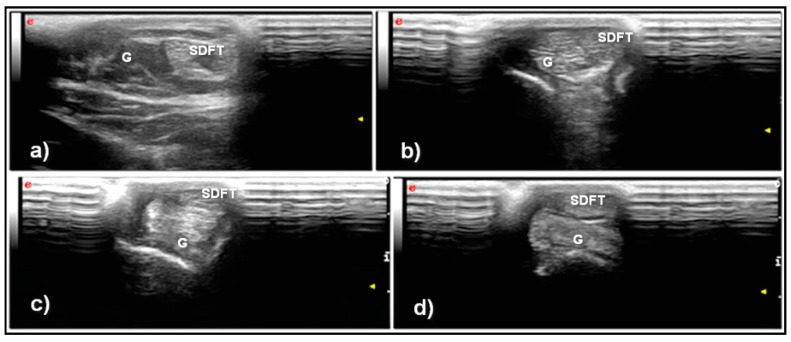
Transverse ultrasonographic scan of the normal ovine CCT: (**a**) myotendinous junction, (**b**,**c**) middle part and (**d**) insertion on the calcaneal tuberosity. Note that toward the distal part, the superficial digital flexor tendon (SDFT) moves to a more superficial position than the gastrocnemius tendon (G).

In the transverse plane, the peritenon of the superficial digital flexor tendon and gastrocnemius appeared as a hyperechoic line with the tendon fibers characterized by a punctiform hyperechoic texture (Figure 2).

**Figure 3:**
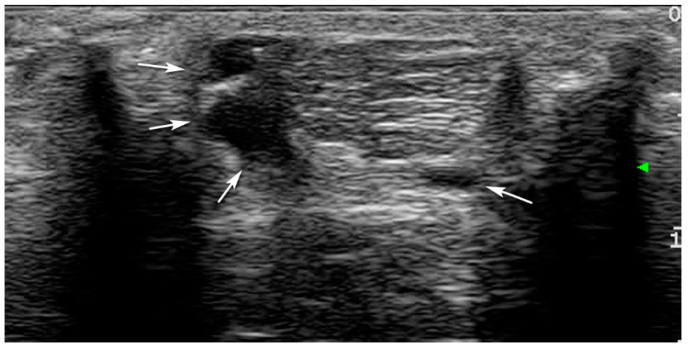
Ultrasonographic features of the induction of the lesion with an injection within the peritendinous space under ultrasound guidance (arrows).

**Figure 4:**
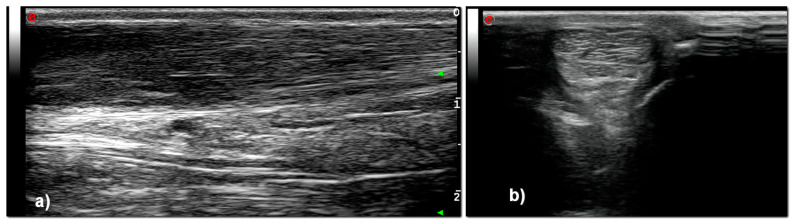
Ultrasonographic appearance of CCT after induction of the lesion, in longitudinal (**a**) and transverse (**b**) scan. The tendon is thicker, bowed and hypoechoic, with abnormal fibrillary architecture.

Excluding T_0_, at all-time points, the entirety of the CCT showed bowing of the tendon with hypoechoic areas, and disruption of normal fiber pattern and were all classified as Grade 3 (Figure 3 and Figure 4). Calcaneal bursa remained normal at each time point.

Clinically assessed width was significantly increased between T_0_ and T_1–2_ and significantly decreased between T_1–2_ and T_3_. No statistically significant difference was present between T_0_ and T_3_ and T_1_ and T_2_.

In the transverse sonograms, the thicknesses of the CCT was significantly increased between T_0_ and T_1_ and significantly decreased between T_1_ and T_2–3_. No differences between T_0_ and T_2–3_ and T_2_ and T_3_ were seen. In the transverse sonograms, the width of the CCT was significantly increased between T_0_ and T_1–2_ and significantly decreased between T_1–2_ and T_3_.

No statistically significant difference was present between T_0_ and T_3_ and T_1_ and T_2_. Data from each time point are reported in Table 1.

There was no statistically significant difference between the clinical and ultrasonographic assessment of tendon width at each time point.

Elastosonography of the CCT was considered to be an easy technique to perform in the longitudinal plane with a good ICC (0.68) when performed by a radiologist. Transverse sonograms were considered difficult to acquire and were frequently characterized by the presence of artefacts. Longitudinal elastogram of the CCTs of sound sheep showed a high percentage of hard tissue, with a mean +\− SD Elx-t%hrd at T_0_ of 89.9% +\− 15.3 and a mean +\− SD Elx-t%sft at T_0_ of 9.9% +\− 15.

**Figure 5:**
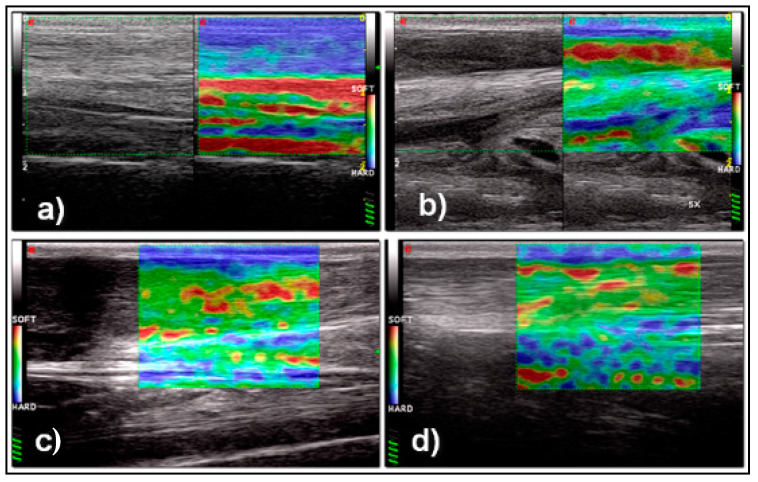
Ultrasonographic and elastosonographic appearance of the normal CCT (**a**): The tendon is homogeneously hard (blue in the color-coded map). Ultrasonographic and elastosonographic appearance of the CCT after 15 days (**b**), 30 days (**c**) and 60 days (**d**) from the induction of the lesion: The tendon is hypoechoic and enlarged with disruption of the normal tendon fibers pattern in all time points. With elastosonography the CCT appears mostly soft (red in color-coded map) after 15 days. After 30 and 60 days the CCT appears of intermediate elasticity (green in color coded map) with areas of softness (red spots on color coded map).

The Elx-t%hrd parameter was significantly higher in normal CCT (T_0_) when compared to T_1–2–3_. No significant differences between T_1_ and T_2_, T_1_ and T_3_ and T_2_ and T_3_ were seen for Elx-t%hrd parameter. The Elx-t%sft parameter was significantly lower in normal CCT (T_0_) when compared to T_1_, T_2_ and T_3_ (Figure 5).

**Figure 6:**
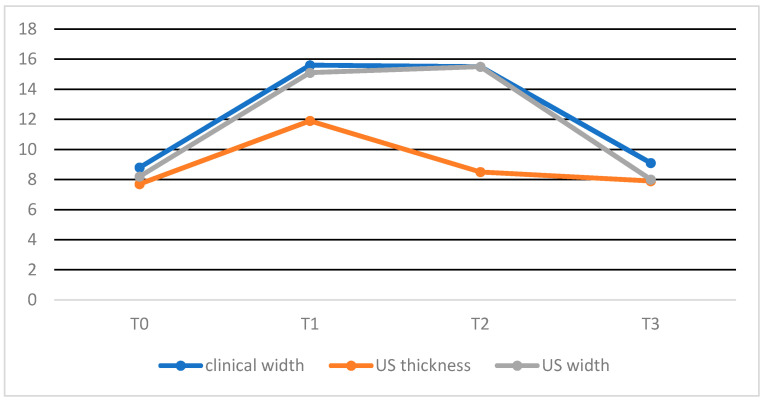
Graphic representation of progression of tendon dimension at each time point, expressed in mm.

**Figure 7:**
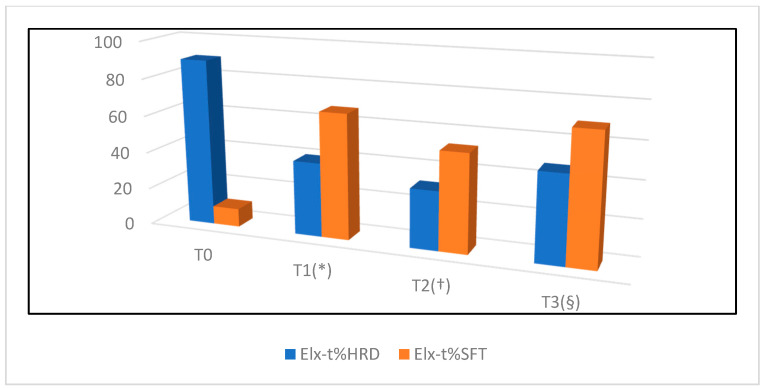
Graphic representation of progression of tendon healing expressed in percentage of hard and soft tissue, assessed by elastosonography. Statistical difference (*p* < 0.05) between: T_0_–T_1_ (*), T_0_–T_2_ (^†^), T_0_–T_3_ (^§^).

No significant differences between T_1_ and T_2_, T_1_ and T_3_ and T_2_ and T_3_ were seen for Elx-t%sft parameter. Data from each time point is presented in Figure 6 and Figure 7.

## 4. Discussion

This prospective study provides preliminary information regarding the ultrasonographic and real-time elastosonographic appearance of the mid-portion of the normal ovine CCT and of the tendon healing process during the first 60 days following creation of an experimentally-induced lesion. As previously described in small animals [29], the ultrasonographic appearance of the mid-portion of the ovine CCT is characterized by a parallel and punctiform hypoechoic echotexture, respectively in longitudinal and transvers sonograms, representative of the organized fibrillar texture of this structure. The superficial digital flexor and gastrocnemius components were easily discernible using ultrasound and delineated by a thick, hyperechoic peritenon. The choice of using collagenases to induce the tendinopathy was based on previous study [30] and was preferred to surgical tenotomy to reduce the costs and the risk of postoperative complications (e.g., surgical site infections). Following injection of collagenases, the ultrasonographic appearance of the tendon changed significantly and was still significantly abnormal (Archambault Grade 3) at the time of the final examination (T_3_). This result reflects the expected evolution of the tendon healing process [31], and confirms the utility of traditional US in the assessment of tendon healing. By 60 days following injury, a tendon is expected to be in an early remodeling phase, in which the fibroblasts, previously migrated from the peritenon, start to remodel into a scar-like tendon tissue and the tendon histologic structure is still severely abnormal [32].

In this study, following intra-tendinous injection of collagenases, the width of the CCT as assessed by manual caliper measurement, as well as the width and thickness as assessed by ultrasound, increased significantly during the first 15 days (T_0_–T_1_) and returned to physiological values at T_3_. These findings are consistent with the initial inflammatory and proliferative stages of tendon healing [31] and with the subsequent complete reabsorption of the edema and hematoma following disruption of the tendon blood vessels. However, while the width of the tendon remained stationary between T_1_ and T_2_, and then reduced significantly between T_2_ and T_3_, the thickness of the tendon reduced progressively from T_1_ to T_3_. The reason for this result is unknown, and possibly, of no clinical relevance. It is possible that the result was influenced by the ultrasound technique itself, as when applying the probe over the CCT region, the operator may have compressed the tendon against the caudal aspect of the distal tibia and reduced the relative tendon thickness. Differences in the pressure applied to the tendon at each time point, may explain this result. Ultrasonographic assessment of CCT healing has been described in dogs and cats [23]. Kramer reported an overall increased tendon diameter within the second and sixth weeks of tendon healing. In Kramer’s study, only after eight weeks did the tendon diameter begin to decrease [23]. However, the study does not report how the tendon diameter was evaluated and does not specify if the tendons were monitored following surgical repair or spontaneous healing of a partial or complete tendon rupture. To the authors’ knowledge, ultrasonographic assessment of Achilles tendon healing, in human medicine, has been only reported following tenotomy and cast application (Ponseti technique) to manage idiopathic congenital talipes equinovarus (clubfoot deformity) in children [33,34,35,36] and following surgical repair to treat Achilles tendon rupture [37]. Trying to compare our results with the aforementioned studies would not be appropriate. Real-time elastosonography of the CCT resulted a subjectively fairly easy technique when performed by a radiologist in the longitudinal plane. Due to the lack of complete contact between the probe and the tissues, problems in the acquisition of reliable and artefact-free sonograms were experienced in the transverse plane. Our findings are consistent with previous humans’ and veterinary studies, as the elastosonographic assessment of the Achilles tendon in longitudinal plane has been proven to be more reproducible than in the transverse plane [38]. Similar findings have been observed in the assessment of the patellar ligament in healthy dogs [18]. Similar to the Achilles tendon in humans, the ROI of the CCTs of sound sheep examined in this study, was characterized by a high percentage of hard tissue [38,39]. Following lesion induction, the ovine CCT showed a severe reduction in stiffness and increased softness during the first 60 days. Our observation is consistent with previous preliminary studies in the human literature on Achilles tendinopathies [38,39,40,41,42,43]. Despite the normalization in tendon width and thickness at T_3_ the CCT continued to show significantly decreased stiffness and increased softness compared to T_0_. The overall percentage in hard tissue in the ROI examined at T_3_ was approximately 50% of the original stiffness of the tendon. The persistent changes in tendon elasticity are consistent with the expected microscopic healing process and the subsequent changes the subsequent changes in mechanical proprieties of the tendon. Even in the long term, tendons heal forming a fibrovascular scar and consequently lose biomechanical elastic properties [38].

To the authors’ knowledge, this is the first longitudinal prospective study that use an ovine model to assess the elastosonographic progression of tendon healing. In the human literature, RTE has been shown to be superior to traditional ultrasound for the diagnosis of Achilles tendinopathy, with superior correlation between the elastosonographic results and functional scores [44]. Moreover, Klauser et al. provided evidence of correlation between histological evidence of pathological changes and a softer tendon elastogram [25]. Yamamoto et al. has demonstrated that RTE correlate with histologic and mechanical proprieties of tendon healing in a rabbit model [12]. This experimental ovine model provides further evidence in support of the use of RTE for the assessment of tendon elasticity following a tendon injury. The use of RTE may be considered during the recovery from CCT tendon injury, for example to modulate rehabilitation or to guide ongoing treatment according to the evolution of the tendon elasticity.

This study has several limitations including the low number of sheep included and the absence of a control group. The contralateral CCT could have been used as control group, however, these data were collected as part of a larger study and it was not possible to use the contralateral CCT as a control. Considering the severe loss of stiffness at T_3_, a longer follow-up would have provided more information regarding progression of the mechanical proprieties of the CCT. The localization chosen for the injection of the collagenases, and the fact that only this specific ROI of the tendon was assessed, could be considered as possible limitations. It is possible that our results might not be representative of tendon lesions at level of its insertion into the calcaneal tuberosity nor at level of the musculotendinous junction. Moreover, the “tendinopathy model” used in this study might not be adequate to represent different natural occurring diseases, such as chronic degenerative tendinopathies.

In conclusion, this study describes the normal ultrasonographic appearance of the mid-portion of the common calcaneal tendon in healthy sheep. This study provides evidence that following an experimentally-induced tendon lesion, despite restoring of physiological thickness and width, ultrasonographic and elastosonographic changes in the ovine CCT persist for at least 60 days. Tendon thickness and width evaluation may not appropriately reflect restored mechanical function of the tendon. Additional studies are required to assess the long-term evolution of tendon healing and the correlation between the ultrasonographic appearance of the tendon and its mechanical proprieties, as evaluated with real-time elastosonography.

## Figures and Tables

**Table 1:** Mean +/− SD of clinical, ultrasonographic and elastosonographic tendon assessment, at each time point.

	T_0_	T_1_	T_2_	T_3_
**Mean +/− SD clinical CCT width**	8.8 +/− 1.6 mm	15.6 +/− 1.6 mm (*)	15.5 +/− 2.3 mm (^†^)	9.1 +/− 1.1 mm (^x,^^‡^)
**Mean +/− SD US CCT thickness**	7.7 +/− 2.0 mm	11.9 +/− 2.9 mm (*)	8.5 +/− 1.9 mm (°)	7.9 +/− 0.8 mm (^x^)
**Mean +/− SD US CCT width**	8.2 +/− 1.6 mm	15.1 +/− 1.6 mm (*)	15.5 +/− 1.6 mm (^†,^°)	8.0 +/− 1.5 mm (*^,x^)
**Mean +/− SD Elx-t%hrd**	89.9 +/− 15.3	40 +/− 25.1 (*)	31.8 +/− 19 (^†^)	47.2 +/− 29.1 (^§^)
**Mean +/− SD Elx-t%sft**	9.9 +/− 15	67.4 +/− 26.4 (*)	52.8 +/− 29.5 (^†^)	69.6 +/− 19.2 (^§^)

Statistical difference (*p* < 0.05) between: T_0_–T_1_ (*), T_0_–T_2_ (^†^), T_0_–T_3_ (^§^), T_1_–T_2_ (°), T_1_–T_3_ (^x^) and T_2_–T_3_ (^‡^). Elx-t%hrd: percentage of hard tissue within the tendon / Elx-t%sft: percentage of soft tissue within the tendon.

## Data Availability

The Data presented in this study are available within the article.
